# Celastrol: A Spectrum of Treatment Opportunities in Chronic Diseases

**DOI:** 10.3389/fmed.2017.00069

**Published:** 2017-06-15

**Authors:** Rita Cascão, João E. Fonseca, Luis F. Moita

**Affiliations:** ^1^Instituto de Medicina Molecular, Faculdade de Medicina, Universidade de Lisboa, Lisbon, Portugal; ^2^Rheumatology Department, Centro Hospitalar de Lisboa Norte, EPE, Hospital de Santa Maria, Lisbon Academic Medical Centre, Lisbon, Portugal; ^3^Instituto Gulbenkian de Ciência, Oeiras, Portugal

**Keywords:** celastrol, inflammation, cancer, neurodegenerative diseases, toxicity, treatment, clinical trials

## Abstract

The identification of new bioactive compounds derived from medicinal plants with significant therapeutic properties has attracted considerable interest in recent years. Such is the case of the *Tripterygium wilfordii* (TW), an herb used in Chinese medicine. Clinical trials performed so far using its root extracts have shown impressive therapeutic properties but also revealed substantial gastrointestinal side effects. The most promising bioactive compound obtained from TW is celastrol. During the last decade, an increasing number of studies were published highlighting the medicinal usefulness of celastrol in diverse clinical areas. Here we systematically review the mechanism of action and the therapeutic properties of celastrol in inflammatory diseases, namely, rheumatoid arthritis, systemic lupus erythematosus, inflammatory bowel diseases, osteoarthritis and allergy, as well as in cancer, neurodegenerative disorders and other diseases, such as diabetes, obesity, atherosclerosis, and hearing loss. We will also focus in the toxicological profile and limitations of celastrol formulation, namely, solubility, bioavailability, and dosage issues that still limit its further clinical application and usefulness.

## Introduction

**Figure 1:**
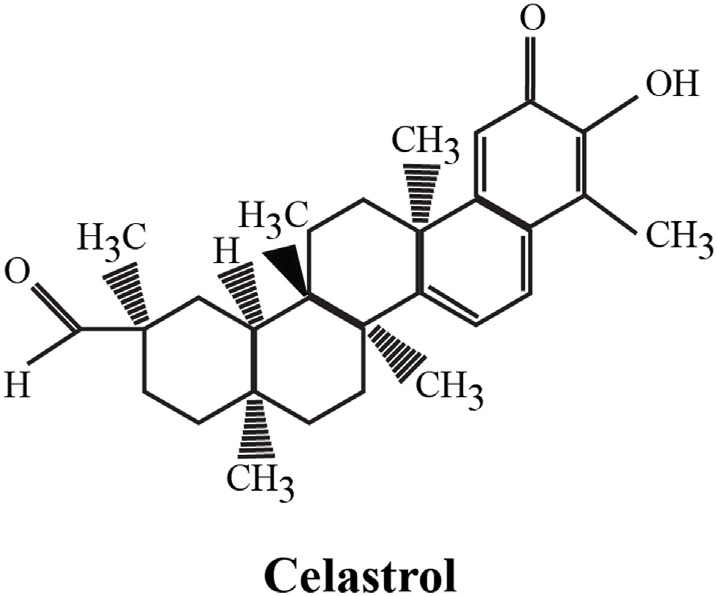
Chemical structure of celastrol. The chemical formula of celastrol is C_29_H_38_O_4_ [adapted from Ref. (1)].

The identification of bioactive compounds derived from medicinal plants has attracted considerable interest in recent years for their strong and in some cases unique, anti-inflammatory, anticancer, and neuroprotective properties. One representative example is the *Tripterygium wilfordii* (TW) plant, used in Chinese medicine to treat an array of immunological disorders, including rheumatoid arthritis (RA), with promising results in a series of clinical trials. TW is a perennial vine of the *Celastraceae* family, also called Thunder God Vine or “lei gong teng” (Chinese name). This plant is poisonous but its root pulp contains several therapeutic compounds, including terpenoids, alkaloids, and steroids. The chemical structure of its purified compounds has been determined by nuclear magnetic resonance and mass spectroscopy. More than 46 diterpenoids (such as triptolide), 20 triterpenoids (e.g., celastrol), 21 alkaloids (like euonine), and other small molecules have been identified from TW. The most abundant and promising bioactive compound derived from the root of this plant is celastrol, also called tripterine, which possess a broad range of biological activities. Celastrol (3-hydroxy-9β,13α-dimethyl-2-oxo-24,25,26-trinoroleana-1(10),3,5,7-tetraen-29-oic acid) is a pentacyclic triterpenoid (Figure 1) that belongs to a small category of natural products of triterpene quinine methides.

Here, we systematically review the therapeutic properties of celastrol in chronic diseases and its toxicological profile, illustrating its potential clinical application.

## Clinical Experience with the Use of TW

Clinical trials using TW plant extracts have already been conducted in inflammatory diseases.

TW is generally used in the treatment of Crohn’s disease (CD) in China. Its therapeutic benefits have been explored in an open-label clinical trial conducted in 20 CD patients treated with TW tablets (120 mg daily) for a period of 12 weeks. CD Activity Index diminished during the first 8 weeks, and the endoscopic improvement was observed after 12 weeks. Inflammatory parameters, including c-reactive protein (CRP), also decreased (2). In addition, two placebo-controlled trials and one prospective single-blind clinical trial have studied the therapeutic potential of polyglycoside TW (1 mg/kg daily) in the prevention of postsurgical relapses in patients with CD. Results from these studies suggest that this is an effective and well-tolerated drug (3–5). Recently, a randomized clinical trial has shown that TW (1.5 mg/kg/day) was comparable to azathioprine to prevent postoperative clinical recurrence of CD, although less efficient in preserving endoscopic remission at week 52 (6). An additional clinical trial enrolled 198 patients with CD, which were randomized to receive mesalazin (3 g/day), low-dose TW (1.5 mg/kg/day) or high-dose TW (2.0 mg/kg/day) over a 52-week period (7). Importantly, data have shown that less patients in the high-dose group (7/71) had clinical recurrence in comparison with patients in the low-dose (15/68, *p* = 0.047) or patients treated with mesalazin (17/59, *p* = 0.006). However, patients under mesalazin treatment had less adverse effects than those treated with high-dose (*p* = 0.029) and low-dose of TW (*p* = 0.048) (7).

The benefits of TW plant extracts have also been tested in psoriatic patients. A randomized clinical trial has shown equal efficacy of TW (20 mg, three times a day) and acitretin for the treatment of *psoriasis vulgaris* for 8 weeks in a total of 115 patients (8).

Additionally, several clinical trials with TW have already been conducted in RA. Uncontrolled trials from 1980s enrolling more than 100 RA patients have shown 87% response rates. To evaluate these claims, in 2002, a study recruited 35 patients and randomized them to placebo, low (180 mg/day) or high (360 mg/day) dose of an ethanol/ethyl acetate extract of TW (9). After 5 months, 80% of the high-dose and 40% of the low-dose groups had achieved an American College of Rheumatology 20% (ACR20) improvement response criteria, compared with none of the patients taking placebo. Both physical function and inflammation were improved. Diarrhea was the most common adverse event followed by nausea. A systematic review of a total of seven randomized controlled trials was done in 2009 by Jiang and colleagues to evaluate the efficacy and safety of TW in the treatment of RA (10). An interesting conclusion was that although TW was clinically as effective as disease-modifying anti-rheumatic drugs (DMARDs), no effect was observed in delaying bone erosions. However, these are small sample size trials and in six of them there were some methodological limitations that can have produced relevant biases. Subsequently, Goldbach-Mansky’s group enrolled 121 RA patients and randomly assigned them to receive TW extract (60 mg, three times daily) or sulfasalazine (11). After 6 months, 68% of those treated with TW and only 36% of those under sulfasalazine reached an ACR20 response. More patients in the sulfasalazine group experienced moderate or severe adverse effects. The most frequent side effects in patients receiving TW were diarrhea, nausea, dyspepsia, abdominal pain, and upper respiratory tract infection (11). In 2012, an interesting concept was tested in a randomized controlled, single-blind clinical trial that used external application of TW extracts in 67 active RA patients with positive results and only referring two cases of mild skin allergy (12). This result has been also reported by Cibere et al. (13). In 2013, due to inconsistency in some clinical trial data, Liu et al. performed a meta-analysis of randomized controlled trials. In this review, authors have concluded that TW extracts are a sort of "herbal DMARD" with a similar efficacy to synthetic DMARDs in RA treatment and that well-designed confirmatory randomized controlled trials should be done (14). Recently, Zhang and co-workers have shown in a multicenter, open-label, randomized controlled trial that combined therapy of methotrexate (MTX) and TW was more effective than MTX monotherapy in the treatment of active RA patients (15, 16). In this study, about 52.7% of the 207 patients experienced adverse events, which were seen in 46.4, 62.3, and 49.3% of patients receiving TW (20 mg, three times per day), MTX (12.5 mg/week), and TW + MTX, respectively (*p* = 0.136). The most common adverse event was mild gastrointestinal side effect, reported by 43.5% of patients on MTX and by 34.8% of those receiving the combination (16). According with this reported experience, combining both medications might be a good strategy to decrease the amount of MTX needed, which may reduce the toxic effects that can limit MTX long-term treatment (17). This report is in accordance with an observation from 2001, where 70 RA patients receiving MTX combined with small doses of TW polyglycoside (10 mg, three times a day) had a better effect and less adverse reactions than monotherapy with MTX (18). Finally, an interesting clinical trial has been conducted in order to evaluate the efficacy and safety of etanercept plus TW (10 mg, three times per day) in elderly patients with active RA. Etanercept plus TW had an equivalent therapeutic effect to that of Etanercept plus MTX and were both well tolerated (19). Altogether, these clinical trials show relevant data regarding the use of TW in RA treatment; however, there are limitations in their design, including the open-label design, short duration (6 months), and lack of radiological assessment. Although TW extracts showed efficacy in the symptomatic treatment of RA patients, it is still unclear if it provides structural damage control.

Regarding the clinical experience with TW in cancer, two water-soluble derivatives of triptolide (TW diterpenoid, a different class from celastrol) have been synthesized (PG490-88 and F60008) and approved for entry into a Phase I clinical trial for the treatment of solid tumors. PG490-88 will be tested in a Phase I clinical trial for prostate cancer in USA (20). Also, a phase I trial was performed with F60008 given intravenously in 20 advanced solid tumors patients in a total of 35 cycles. The most frequent side effects were mild anemia, fatigue, nausea, vomiting, diarrhea, and constipation. Two lethal events were observed and the high inter-individual variability rendered this derivative far from optimal (21).

In the case of neurodegenerative disorders, no clinical data are available about the use of TW. A study regarding the use of celastrol in amyotrophic lateral sclerosis concludes that further preclinical data, human toxicity, and pharmacokinetic results are required to proceed with trials (22).

The therapeutic effect of TW extracts has also been evaluated in diabetic patients. A prospective clinical trial enrolled 45 patients with type 2 diabetic kidney disease, randomly divided into three groups: TW (1–2 mg/kg/day), irbesartan (150–300 mg/day), and TW combined with irbesartan. Data have shown that treatment with TW for 12 weeks may be effective in preventing podocyte injury with a synergistic protective effect with irbesartan (23). Another clinical trial has been performed to evaluate the efficacy of TW in the treatment of type 2 diabetes mellitus (DM)-induced nephropathy. A total of 65 patients were enrolled in this 6-month, prospective, controlled study, and randomized into treatment groups: 120 mg/day of TW extract for 3 months, followed by 60 mg/day for 3 more months, or 160 mg/day of valsartan for 6 months. It was found that TW can significantly reduce the urine protein levels (24). Similar nephroprotective effects for TW preparations have been described in a meta-analysis of randomized controlled trials of chronic kidney disease patients (25).

A clinical trial, in China, also tested the use of TW in human kidney transplantation (26). Rejection occurred in 4.1% of patients treated with TW versus 24.5% of control patients, showing efficacy in the prevention of renal allograph rejection. All patients tolerated well TW administration during the 5 years of the study (26). This interesting potential of TW, which was already demonstrated in different *in vitro* and *in vivo* experimental set-ups, may be of interest in several medical areas.

In summary, clinical trials have only tested TW plant extracts. Despite its potential clinical usefulness, the sale of TW has been prohibited in many countries because the misuse of the herb can cause severe consequences, including diarrhea, nausea, and infertility. Even an apparent non-toxic dose may cause antifertility effects in men, male rats, and guinea pigs due to oral administration of some toxic components of TW extracts. Unfortunately, in men, this dose is only one-third of the recommended dose for the treatment of RA or skin diseases (27–29). As such, treating patients with TW bioactive compounds with known pharmacological properties may circumvent toxicological limitations. As stated before, celastrol is the most abundant bioactive compound existing in TW and these impressive therapeutic properties support the growing interest on this compound.

## Anti-Inflammatory Properties of Celastrol

The therapeutic usefulness and anti-inflammatory properties of celastrol have been studied in several inflammatory diseases, including RA, ankylosing spondylitis, systemic lupus erythematosus (SLE), inflammatory bowel disease, osteoarthritis (OA), allergy, and skin inflammation.

### Rheumatoid Arthritis

**Figure 2:**
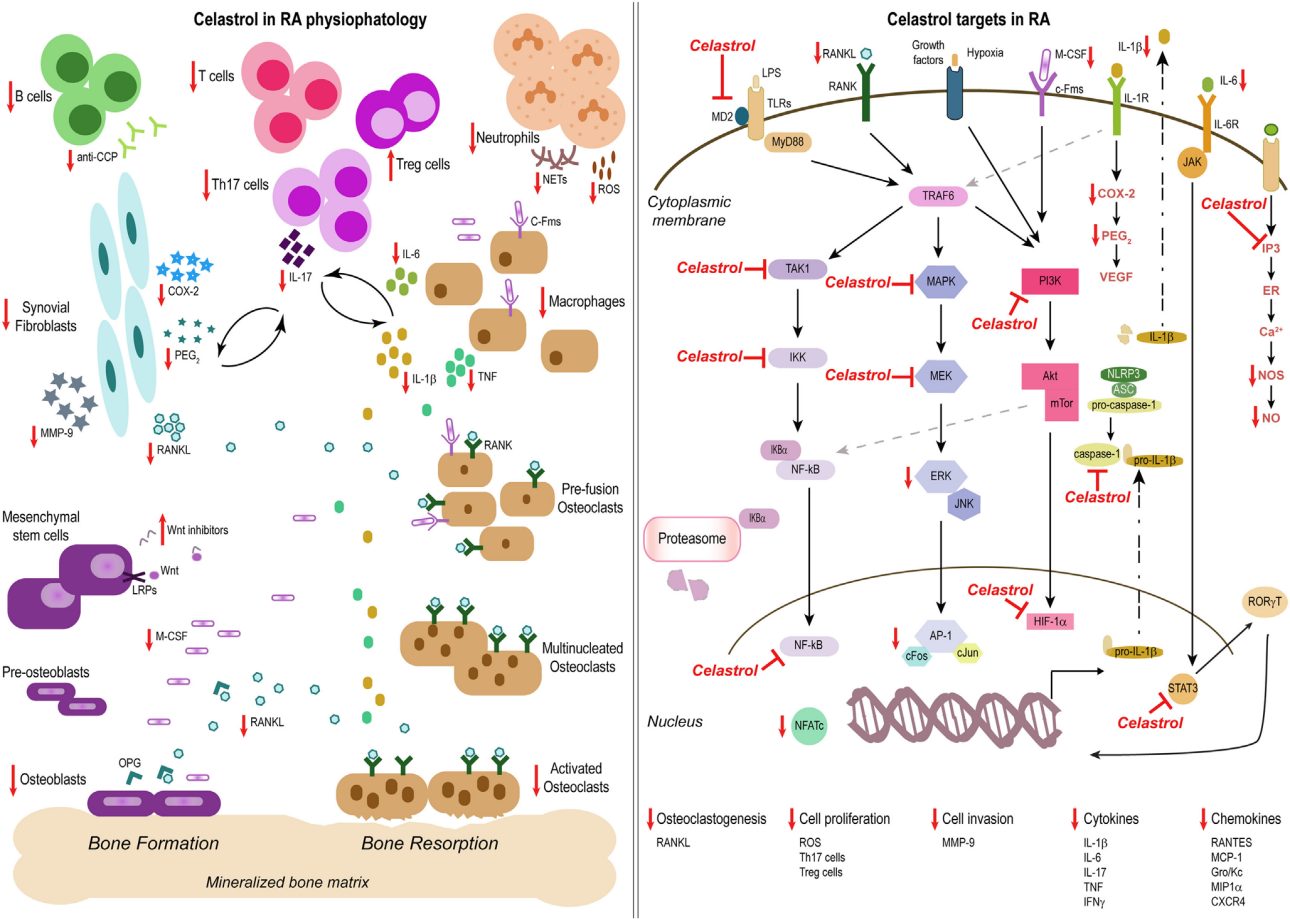
Celastrol in the treatment of rheumatoid arthritis (RA). Schemes illustrate the main anti-inflammatory properties and molecular targets of celastrol in the physiopathology of RA, as a prototype of an inflammatory disease. Celastrol has several cellular targets, interfering with the production of cytokines, chemokines, and inflammatory mediators; inhibiting cell invasion and proliferation; and suppressing bone resorption and thus constitutes a potential candidate for the treatment of inflammatory diseases.

RA is a chronic inflammatory immune-mediated disease characterized by polyarthritis and joint damage. Both immune cells and cytokines play crucial roles in the pathogenesis of this disease. The anti-inflammatory properties of celastrol in this condition have so far been attributed to the: (i) regulation of cytokine and chemokine production, (ii) regulation of inflammatory mediators expression; (iii) modulation of inflammatory cell functions, and (iv) osteoclast modulation and bone damage control (Figure 2).

#### Regulation of Cytokine, Chemokine, and Inflammatory Mediators’ Production

In 1991, Xu et al. (30) have shown *in vitro* that tripterine (celastrol) inhibits lipopolysaccharide (LPS)-induced interleukin (IL)-1β production from murine peritoneal macrophages and human monocytes, concanavaline A-activated IL-2 production by murine splenocytes, and prostaglandin E_2_ (PEG_2_) release from synovial cells. Moreover, *in vivo* it possess disease-modifying activities in streptococcus cell wall-induced (SCW) and collagen-induced (CIA) arthritis mouse models (31). Accordingly, Li et al. (32) have reported that tripterine inhibits paw swelling and bone destruction in adjuvant-induced arthritis (AIA) rats associated with a decrease in IL-1β mRNA in the synovial membrane and tumor necrosis factor (TNF) mRNA in hind paw tissue. Later, *in vitro* data have revealed that low concentrations (0.05–1 μM) of celastrol inhibit the production of nitric oxide (NO) and PEG_2_, accompanied by the decrease in iNOS and cyclooxygenase (COX)-2 protein, inhibit TNF and IL-6 release, and suppress the activity of nuclear factor kappa B (NF-kB) and activator protein (AP)-1 in LPS-stimulated macrophages (33, 34). *In vivo*, Venkatesha et al. (35) have shown that celastrol possess antiarthritic activities in AIA rats. It inhibits IL-6, IL-17, and interferon (IFN)-γ mRNA in lymph node (LN) and spleen adherent (SAC) cells isolated from arthritic rats. In LN cells celastrol induces a reduction in pSTAT3 but not in RORγt, suggesting that it may interfere with IL-17 production by T helper (Th)17 cells rather than with Th17 cells differentiation. In addition, celastrol reduces IL-6 production, metalloproteinase (MMP)-9 and pERK in AIA-derived fibroblasts, with no effect on VEGF and MMP-2. Interestingly, it also reduces anti-CCP serum levels in AIA rats (35). The same group has reported that celastrol inhibits the production of proinflammatory cytokines (TNF and IL-1β) and chemokines (RANTES, MCP-1, and GRO/KC) by SAC cells isolated from AIA rats after treatment. It also inhibits the migration capability of these cells (36). Except for MCP-1, serum levels of these cytokines and chemokines were also decreased in celastrol-treated AIA rats. Additionally, the same results, together with a reduction in MIP-1α, were observed in synovial fibroblasts isolated from arthritic rats and cultured with celastrol. Regarding chemokine receptors, celastrol does not seem to have much effect on their cellular expression, besides CCR1 (36). Of note, CCR1 correlates with the level of inflammation in experimental arthritis (37, 38).

Accordingly, in 2012 our group showed that celastrol decreases the secretion of both IL-1β and TNF in the THP-1 macrophage-like cell line, associated not only with NF-kB inhibition but also with caspase-1 inactivation (39). *In vivo*, we observed that celastrol has anti-inflammatory effects both in an early and established phase of arthritis development, suppressing ankle swelling, joint inflammatory cell infiltration and proliferation, and serum IL-6 levels in AIA rats (39, 40). This inhibitory effect in cellular infiltration and proliferation may prevent and treat synovial pannus tissue development characteristic of RA patients and responsible for bone damage.

Recently, it was found that celastrol inhibits LPS-induced cytokine production in macrophages by blocking LPS binding to MD2 from the MD2/TLR4 complex, inhibiting TLR4 signaling activation and thus the initiation of inflammatory responses (41).

This ability of celastrol to suppress the production of cytokines and chemokines might lead to the inhibition of leukocyte migration into the inflamed joints, contributing to its anti-inflammatory activity.

#### Modulation of Inflammatory Cell Functions

In human fibroblasts isolated from RA patients (RA-FLS), it has been shown that celastrol inhibits LPS-induced migration and invasion by inhibiting MMP-9 expression and activity (42). It inhibits MMP-9 transcription through the suppression of the binding activity of NF-kB in the promoter and the TLR4/MyD88/NF-kB pathway signaling (42). Previously, these authors have also reported that celastrol inhibits IL-17-induced migration and invasion of human RA-FLS by suppression of NF-kB-mediated MMP-9 expression and activity (43). In addition, under hypoxic conditions mimicking the synovial hypoxia of arthritic joints, it has been found that celastrol decreases hypoxia-induced FLS invasion by inhibiting HIF-1α-mediated CXCR4 transcription (44).

In 2015, our group has reported that celastrol restores synovial homeostasis in AIA rats, with a reduction in joint synovial CD3+ and CD19+ lymphocytes, associated with a suppression of bone erosions and a remarkable reduction in CD68+ macrophages, a marker of therapeutic efficacy in human RA and experimental arthritis. Importantly, neither blood biochemical parameters nor tissue histological structures revealed drug-induced toxicity associated with the intraperitoneally administration of 1 mg/kg/day of celastrol for 18 days (40).

Interestingly, Yu et al. have shown *in vitro* that non-cytotoxic concentrations (≤10μM) of celastrol inhibit neutrophil oxidative burst and extracellular trap (NET) formation through the inhibition of SYK/MEK/ERK and IkBα signaling cascade (45). Moreover, authors have shown that it decreases the levels of citrullinated histones, which are autoantigens in RA patients (45). Recently, Astry and colleagues have described, in synovium-infiltrating cells (*SIC*) from AIA rats, that celastrol reduces Th17 and increases T regulatory (Treg) cell frequencies, possibly favoring an anti-inflammatory/immunomodulatory local environment in the inflamed joints (46). *In vitro*, it inhibits Th17 and promotes Treg differentiation through the decrease of pSTAT3 as well as of IL-1β and IL-6 production (46). These data, together with our results showing that celastrol is more effective than Digoxin (specific RORγt inhibitor and hence suppressor of Th17 cells differentiation) in suppressing arthritis in AIA model (39), suggest that the combined effect of celastrol in both Th17 and Treg cells is an advantage in the treatment of arthritis.

#### Osteoclast Modulation and Bone Damage Control

In 2010, Idris et al. have shown in osteoblast/bone marrow co-cultures that celastrol inhibits osteoclast formation and bone resorption, and suppresses IL-1β-induced receptor activator of nuclear factor kappa-B ligand (RANKL) expression in osteoblasts by NF-kB signaling inhibition (47). In bone marrow cultures, it suppresses M-CSF and RANKL-induced osteoclast formation and induces apoptosis, *via* inhibition of NF-kB, ERK, and c-Fos activation. In osteoblasts, it prevents TAK1 activation upstream of NF-kB and reduces cell viability and activity (47). These results suggest that, at least *in vitro*, celastrol suppresses both osteoclastic and osteoblastic activities, affecting not only bone resorption but also bone formation. Nanjundaiah and colleagues have found that celastrol suppresses inflammation and bone erosions in rats with AIA, with an increase of bone volume coupled with a decrease of osteoclast numbers (48). Authors have shown that it decreases the production of IL-1β, IL-6, IL-17, IL-18, and TNF by *SIC* cells harvested from arthritic rats, inhibiting RANKL production and decreasing RANKL/OPG ratio, consequently inhibiting osteoclastogenesis. Also, celastrol reduces MMP-9 production, which limits bone damage (48). More recently, Gan et al. have described that celastrol not only directly suppresses osteoclast formation and function, but also reduces the RANKL-induced expression of osteoclastic genes and transcriptional factors (49). Authors have demonstrated that it inhibits osteoclast differentiation and function in RAW264.7 cells, with a reduction in osteoclastic genes (*MMP-9, Trap*, and *Ctsk*) and in transcriptional factors (c-Fos, c-Jun, and NFATc1), possibly due to NF-kB and MAPK inhibition (49). These results suggest that celastrol acts both downstream of RANKL at the levels of NF-kB activation and upstream targeting proinflammatory cytokine signaling. *In vivo*, these authors have also observed that it suppresses arthritis and ankle joint destruction in CIA mice, with a decrease in osteoclast numbers, as well as in osteoclastic genes and transcriptional factors (49).

Furthermore, data from our lab have revealed that celastrol is able to control inflammation-induced bone damage, with a reduction in articular cartilage degradation and bone erosions (40). In accordance, Liu and colleagues have observed in a mouse model of dexamethasone-induced secondary osteoporosis that celastrol not only improves lipid metabolism and reduces hypercalciuria, but also mitigates articular cartilage lesions, decreases NF-kB, MMP-1, and MMP-9 expression, and reduces serum PTH, tartrate-resistant acid phosphatase (TRACP)5b, CTX-I, as well as deoxypyridinoline (DPD), suggesting that it ameliorates abnormal bone metabolism (50).

In contrast to RA, ankylosing spondylitis (AS) is an inflammatory and autoimmune disease mainly characterized by new bone formation in axial joints (51, 52). PGE_2_ modulates the anabolic/catabolic process of bone, promoting bone remodeling through osteoblastic cell differentiation (53–55). It has been already reported that celastrol suppresses LPS-induced expression of PEG_2_ *via* the downregulation of COX-1 and COX-2 activation (33, 56). Recently, Zou et al. (57) have pointed out *in vitro* that celastrol inhibits the proliferation of PEG_2_-induced AS fibroblasts and their differentiation into an osteogenic phenotype, associated with a decrease in PI3K/Akt pathway and increase in Wnt inhibitors.

Therefore, data show that celastrol is effective in treating persistent synovitis and preventing cartilage and bone damage, which are the hallmarks of RA physiopathology. Remarkably, this bone-protective property of celastrol in arthritic models is further supported by studies performed in cancer models, as described next. Although the three main pharmacological mechanisms of celastrol that contribute to its efficacy are already extensively studied in arthritis, most of the data results from *in vitro* experiments, which may not completely reflect the *in vivo* complexity of the disease. Specifically, the protective property of celastrol against bone damage needs additional research in *in vivo* models with the inclusion of morphologic and mechanical testing.

Finally, the major complaint of patients with RA, or other forms of arthritis, is joint pain derived from joint inflammation and damage. Interestingly, Yang and co-workers have demonstrated in animal models of inflammatory pain that celastrol not only reduces the mRNA expression of IL-1β, IL-6, and TNF in mice paws but also has antihyperalgesic effects, possible mediated by the activation of cannabinoid receptor-2 (CB_2_) signaling (58). CB_2_ inhibits proinflammatory factors release from inflammatory cells near nociceptive neuron terminals, reducing pain perception without centrally mediated side effects (59). This new interesting capability of celastrol should be further investigated in animal models of other diseases because it could be useful as an adjuvant therapy in different medical areas.

### Systemic Lupus Erythematosus

SLE is a chronic autoimmune inflammatory disease that affects multiple organ systems, prototypically characterized by high levels of circulating autoantibodies and glomerulonephritis. In 2003, a study using the spontaneous (NZBxW)F1 mice model of experimental SLE has shown that treatment with 3 and 6 mg/kg/day of celastrol reduces urinary protein excretion and serum anti-ds DNA autoantibodies, ameliorating clinical symptoms and survival rate (60). In agreement, Li et al. have also found in an experimental SLE mice model induced by active chromatin that administration of 12 mg/kg/day of celastrol decreases circulating anti-ss DNA, anti-ds DNA, and IgG antibodies, reduces serum NO and IL-10 production, and improves splenocyte proliferation, associated with amelioration of proteinuria and renal histological changes (61). Of note, these effects were comparable to 5 mg/kg prednisone in the treatment of experimental SLE (61).

### Inflammatory Bowel Disease

CD and ulcerative colitis (UC) are multifactorial chronic relapsing inflammatory bowel diseases (62), both characterized by an imbalance between pro- and anti-inflammatory cytokines (63). In 2004, a work from Pinna and colleagues has evaluated the effect of celastrol in CD patient’s biopsies of inflamed intestinal mucosa and peripheral blood mononuclear cells (PBMCs). Authors have described that celastrol inhibits proinflammatory cytokine production (IL-1β, IL-6, IL-8, and TNF) in LPS-activated PBMCs and biopsies from CD patients, possibly *via* NF-kB and p38 MAPK inhibition (64). In mice models of DSS-induced colitis, it has been found that celastrol ameliorates acute intestinal injury and prevents the loss of intestinal epithelial homeostasis through the reduction of colonic oxidative stress, inhibition of NLRP3-inflammasome and IL-23/IL-17 pathway, reduction of inflammatory cytokines and increase in IL-10 and TNF levels, attenuation of neutrophil infiltration and upregulation of E-cadherin expression (65, 66). It also suppresses necroptosis death of colonic epithelial cells by upregulating caspase-8 and thus inhibiting RIP3/MLKL axis, avoiding the breakdown of the intestinal barrier and the chronic inflammatory process (66). In this study authors have suggested that the unexpected increase in TNF levels may be induced by the LPS-induced-TNF-α-factor (LITAF), independently of NF-kB (66, 67). More recently, it has been shown that celastrol ameliorates experimental colitis in IL-10 deficient mice *via* the upregulation of autophagy of the colon tissue cells by PI3K/Akt/mTOR signaling downregulation (68).

UC is one of the three highest risk factors for developing colorectal cancer. Importantly, a study from Lin et al. have demonstrated in a mice model of UC-related colorectal cancer (AOM/DSS mice model) that celastrol increases survival rate associated with a reduction in colonic neoplasms, prevents the upregulation of oncogenic markers namely, β-catenin, proliferating cell nuclear antigen (PCNA), and dysfunctional p53, inhibits proinflammatory mediators and NF-kB activation and suppresses epithelial mesenchymal transition (EMT), through E-cadherin upregulation and N-cadherin, vimentin, and Snail downregulation (69). The same group has also found that celastrol inhibits cell proliferation in colorectal cancer cell lines and inhibits tumor growth by reversing EMT in colonic xenografts (69). Finally, an interesting lipidomics report unrevealed that celastrol recovers lysophosphatidylcholine and sphingomyelin metabolism of DSS-induced colitis mice, partially by upregulating stearoyl-CoA desaturase-1 (SCD1) (an enzyme responsible for fatty acids desaturation) expression and restoring the altered balance between steric acid- and oleic acid-derived lipid species against proinflammatory signaling (70).

### Osteoarthritis

OA is a multifactorial joint disease characterized by joint cartilage degradation. This pathology results from an imbalance between anabolic and catabolic activities of chondrocytes, with matrix MMPs, COX-2, and iNOS playing a central role in cartilage degradation. Inflammation of the synovium also occurs, though often mild compared to RA, which is primarily an inflammatory condition. This can happen as breakdown products from the cartilage are released into the synovial space and as cells lining the joint attempt to remove them. Interestingly, it has been found in human osteoarthritic chondrocytes that celastrol suppresses the expression of the catabolic mediators, MMP-1, MMP-3, MMP-13, COX-2, iNOS, and HSP90β, possibly through the inhibition of NF-kB activation (71).

### Allergy

Allergic asthma is a common chronic inflammatory disease of the airways of the lung which is characterized by inflammatory cells infiltration in lung tissues, hypersecretion of mucus by goblet cells, obstruction of the lung airway, and higher expression of Th2 cytokines (72). MMPs mediate airway tissue remodeling through regulation of basal lamina integrity and of the infiltration by inflammatory cells (73, 74). In 2004, Liu et al. have shown that celastrol suppresses airway inflammation in an allergic asthma mice model through the inhibition of histamine and eotaxin production in mast cells (75). Later, in the same animal model, it has been found that celastrol suppresses the ovalbumin-induced airway inflammation through the decrease in airway hyper responsiveness, inflammatory cell infiltration and tissue remodeling. In addition, celastrol also regulates the imbalance of MMP-2, MMP-9, and tissue inhibitor of metalloproteinase 1 and 2 (TIMP-1 and TIMP-2), which is caused by inflammatory cytokines produced *via* MAPK/NF-kB pathway in inflammatory cells (76). Furthermore, *in vitro* data have revealed that celastrol regulates the expression of EMT-related proteins, by the inhibition of immunoglobulin Fc epsilon receptor I (FcεRI) signaling, involving protein kinase C (PKC), Rac1, ERK, and HSP90 (77). Also, it reduces skin inflammation in a Nc/Nga mice model of allergic atopic dermatitis and in a Balb/c mice model of skin inflammation, exerting antiallergic effects (77).

Altogether, data have highlighted that celastrol has great potential as an anti-inflammatory compound mainly due to its ability to inhibit the NF-kB pathway, interfering with the production of proinflammatory cytokines and chemokines, and also with inflammatory cells migration, proliferation, and activation.

## Anticancer Properties of Celastrol

Since 2003 several publications have demonstrated the capacity of celastrol to contribute to the treatment of many types of cancer. Data from different tumor cell lines and animal cancer models have suggested that the anticancer properties of celastrol can be attributed to: (i) cell death activation, (ii) angiogenesis inhibition, (iii) treatment and radiotherapy sensitizing action, and (iv) anti-invasive effect.

### Cell Death Activation

Celastrol inhibits cancer cell progression and induces cell death in a broad range of cancer cell lines such as lung, breast, esopharyngeal, glioblastoma, prostate, hepatoma, myeloma, pancreas, colon, liver, melanoma, leukemia, osteosarcoma, and gastric cancer. Namely, it has been found that celastrol induces cell cycle arrest, apoptosis, and autophagy by the activation of reactive oxygen species (ROS)/c-Jun N-terminal kinases (JNK) signaling pathway (78) in osteosarcoma and by downregulation of miR-21 expression (79–81) in gastric cancer cell lines. This negative regulatory effect of celastrol on microRNAs to induce autophagy was described as well in androgen receptor (AR)-positive prostate cancer cells. In these cells it induces autophagy by inhibiting the AR/miR-101 axis (82). Celastrol also suppresses AR signaling due to receptor degradation. This target to AR signaling occurs *via* HSP90 inhibition or calpain activation (83, 84). Interestingly, in AR-negative prostate cancer cells, celastrol is still able to induce autophagy through HIF/BNIP3 activation (85). The inhibition of HSP90 by celastrol occurs due to its capacity to suppress the interaction of HSP90 with its co-chaperone Cdc37 in pancreatic cancer cells (86). It dissociates the HSP90-Cdc37 complex by interacting with Cdc37 (87), potentially inhibiting some oncogenic proteins. Also, Chadli et al. have shown that celastrol inhibits p23, a HSP90 co-chaperon specific for steroid receptors, which is relevant for steroid-mediated cancers (88). Contradicting data propose that in fact it binds directly to the C-terminal region of HSP90α, inducing oligomerization and affecting some of its functions (89).

In addition to autophagy, celastrol is able to eliminate cancer cells by different apoptotic pathways, including (1) upregulation of death receptors in breast and colon cancer, enhancing TNF-related apoptosis-inducing ligand (TRAIL)-induced apoptosis (90, 91); (2) activation of Fas/Fas ligand pathway in non-small-cell lung cancer (92); (3) inhibition of mitochondrial respiratory chain (MRC) complex I, and consequently ROS accumulation inside cancer cells, in non-small-cell lung carcinoma, liver cancer (93), osteosarcoma (94) and hepatocellular carcinoma (95) cell lines; (4) mitochondrial dysfunction and PI3K/Akt/mTOR pathway inhibition in triple negative breast cancer (96), melanoma cells (97) and several other types of cancer (98); (5) reduction in phosphorylated Akt, mTOR, and S6K and increase in AMP-activated protein kinase (AMPK) phosphorylation in gastric cancer cell lines and xenografts (79); (6) AMPK-induced PLK-2 pathway in breast cancer cell line (99); (7) destabilization of the ErbB2 and estrogen receptors in breast cancer cells (100, 101); (8) activation of caspase-dependent and independent pathways in breast cancer cells (102); (9) inhibition of topoisomerase II in HL-60 leukemia cells (103); (10) mitochondrial instability, activation of caspases and downregulation AML1-ETO/C-KIT oncoprotein, thus inhibiting the Akt, STAT3 and Erk1/2 downstream pathways in acute myeloid leukemia (AML) t(8:21) translocation cell line (104); (11) inhibition of STAT3/Janus kinase 2 (JAK2) in hepatocellular carcinoma (105); (12) inhibition of Myb in AML cells, while not affecting normal hematopoietic progenitor cells (106); (13) induction of the unfolded protein response-dependent cell death, endoplasmic reticulum (ER) stress, and PERK-eukaryotic initiation factor 2 (eIF2)–activating transcription factor (ATF4)-C/EBP homology protein (CHOP) signaling in oral squamous cell carcinoma cell lines (107); (14) reduction of GSK3β levels in HeLa cells (108); (15) target proteostasis in human glioblastoma cells, potentiating the proteotoxic stress response of HSP inhibitors (109); (16) targeting AR, ERG, and NF-kB signaling pathways in prostate cancer (110, 111); (17) upregulation of miR-146a expression, suppressing the NF-kB activity in gastric cancer (112); (18) inhibition of NF-kB in multiple myeloma (113–115), prostate cancer (116, 117) and leukemia cells (118); and (19) downregulation of *IL-6* gene expression *via* NF-kB inhibition in prostate carcinoma cells (119).

As in inflammatory diseases, one key target of celastrol in cancer physiopathology is the NF-kB pathway. It has been demonstrated in prostate cancer, *in vitro* and *in vivo*, that celastrol can induce proteasomal inhibition. This inhibition leads to the accumulation of ubiquitinated Ikβ-α proteins that sequester the cytoplasmic components of the NF-kB complex, consequently causing apoptotic cell death (116, 120). This ability of celastrol to inhibit NF-kB was also observed in breast cancer xenografts, with a tumor growth decrease of approximately 60% (34). It could also augment the apoptotic effects driven by TNF and chemotherapeutic agents, through the suppression of both inducible and constitutive NF-kB activation (34).

In addition to autophagy and apoptosis, celastrol may also induce paraptosis, another form of programmed cell death. The inositol trisphosphate receptor (IP3R)-mediated release of Ca^2+^ from the ER induced by celastrol and its subsequent mitochondrial Ca^2+^ uniporter-mediated influx might lead to a major expansion of mitochondria and ER, and thus to paraptotic cell death. This mechanism has been already descried in breast and colon cancer cell lines (121).

### Angiogenesis Inhibition

Besides cell death, the anticancer properties of celastrol can be also attributed to tumor angiogenesis inhibition. The inhibitory effect of celastrol on angiogenesis is mediated by the suppression of HIF-1α, through HSP90 (122) and mTOR/p70S6K/eIF4E pathway inhibition and ERK1/2 phosphorylation (123, 124). This inhibition of HIF-1α leads to the decrease of its target genes, such as the *VEGF*, that are crucial in the angiogenic process. Nonetheless, it has been shown in human glioma *in vitro* and in xenografts that celastrol does not seem to have an effect on VEGF, but rather in the expression of VEGF receptors (125, 126). On the contrary, a recent study has demonstrated that short-time exposure to celastrol did not alter HIF-1α mRNA levels. Alternatively, it induces HIF-1α protein accumulation in different cancer cell lines in an oxygen-independent manner *via* ROS and Akt/p70S6K signaling activation, promoting the transcription of *VEGF* and *Glut-1* genes (85). These results are contradictory and raise concerns of radiotherapy resistance triggered by low-dose radiation-induced HIF-1α (127, 128). As described above, data also seems to support the inhibitory effect of celastrol upon HIF-1α in the context of arthritis.

### Treatment and Radiotherapy Sensitizing Action

Another interesting anticancer mechanism of celastrol is its radiosensitizing effect. Specifically, celastrol can overcome tumor resistance to radiotherapy in prostate (129) and lung cancer cells (130, 131). This radiosensitizing effect was correlated with significant decreases in HSP90 clients, including epidermal growth factor receptor (EGFR), ErbB2, and survivin, and with an increase in p53 (130). The enhanced cytotoxic effects of ionizing radiation induced by celastrol on human lung tumor cells were also mediated by ROS production (131). Moreover, Wang and colleagues have found that incubation with celastrol after chemo-drug exposure causes persistent DNA damage and apoptosis of lung cancer cells (132). This data was confirmed *in vitro* and *in vivo* using the PC-3 human prostate cancer model (129), in which celastrol induced a reduction of DNA repair capacity, thus enhancing therapeutic efficacy. Recently, results from an *in vitro* study in non-small-cell lung cancer indicate a potential increase in treatment sensitivity due to celastrol-mediated ATF2/cJUN inhibition (133). In leukemia cells, a study has highlighted the ability of celastrol to induce chemotherapy sensitization, associated with caspase-3 activation, PARP cleavage, and decrease in oncoprotein Bcr-Abl (134). Likewise, it depletes Bcr-Abl protein and induces mitochondrial-dependent apoptosis in imatinib-resistant chronic myelogenous leukemia cells (135). Also, in temozolomide-resistant melanoma cells, combined therapy with celastrol has increased treatment sensitization, increasing cell death possibly *via* NF-kB and MAPK pathways (136). Additionally, in some types of cancer, including lung, hepatocellular, and breast cancer, treatment resistance may be overcome by celastrol mainly due to its proapoptotic properties (100, 137–139).

### Invasion Inhibition

Finally, studies have also suggested that celastrol inhibits tumor invasion. In lung adenocarcinoma cells, it has been shown that this compound inhibits TNF-induced invasive activity, which was correlated with the downregulation of NF-kB-mediated gene products and inhibition of MMP-9 (140). Similar results were also described in breast cancer cells (141). This enzymatic breakdown of the extracellular matrix constituents by MMPs is one critical early step for the metastatic process. In accordance, a study published by Mi et al. have demonstrated that celastrol induces breast tumor cells apoptosis and inhibits their invasion *via* downregulating TNF-induced MMP-9 expression, with no effect on MMP-1 and MMP-2 (142). Additionally, it has been suggested that, apart from NF-kB inhibition, celastrol also inhibits invasion of hepatocellular carcinoma cells through the reduction of miR-224 expression, decreasing MMP-2 and MMP-9 protein levels (143). More recently, data have proposed that the inhibition of metastasis in lung cancer cells occurs through the suppression of Akt signaling pathway and integrin expression (144). Also, in esophageal cancer cells, the antimetastatic effect of celastrol was attributed to the inhibition of Wnt signaling pathway and integrin expression (145). The role of integrin inhibition in this process was further pointed out in melanoma cancer cells. In these cells the inhibition of migration and invasion by celastrol was attributed to the regulation of integrin function and cell adhesion, partly *via* p38 MAPK activation (146). Likewise, in colon and pancreatic cancer cells, celastrol reduces tumor invasiveness by downregulation of the chemokine receptor CXCR4 expression (147). Importantly, Idris et al. have proven that celastrol can prevent osteolytic bone metastasis, inhibiting osteoclastic formation and survival mainly due to the suppression of TAK1 and IkappaB kinase (IKK) complex activation (148).

Collectively these reports demonstrate that celastrol has potential to be used in the treatment of different types of cancer, mainly due to its capacity to inhibit transcription factors, such as NF-kB and HIF-1α, and mediators of protein homeostasis, like HSP90 chaperon. Moreover, the ability of celastrol to pass the brain blood barrier makes this compound an attractive therapeutic option in brain tumors and brain metastasis, which are associated with poor prognosis due to ineffective treatments and long-term toxicity.

## Neuroprotective Properties of Celastrol

In the last decade, some studies have reported that celastrol is a promising neuroprotective agent in animal models of neurodegenerative diseases, such as Parkinson disease (149), Huntington disease (149–151), Alzheimer disease (152), and amyotrophic lateral sclerosis (153–155). Neurodegenerative diseases have been termed “protein misfolding disorders” and are characterized by the neuronal accumulation of protein aggregates (156–158). HSPs are repair agents which provide a line of defense against these misfolded, aggregation-prone proteins (158). Importantly, celastrol leads to the induction of HSPs (159). It is capable of inducing a set of HSPs in differentiated neurons in humans and in rodent cell lines (160). Interestingly, celastrol induces a wider set of potentially neuroprotective HSPs, including HSP70B’, in differentiated human neurons compared to differentiated rodent neurons (160), which may suggest that it confers greater protective effects against neurodegenerative diseases in the human brain (149, 150, 153). The induction of HSP70 confers several important therapeutic benefits: (i) maintain cellular protein quality status; (ii) inhibit inflammatory responses by binding to the regulatory NF-kappa-B essential modulator (NEMO) unit in the IKK complex, reducing its activation (161, 162); and (iii) bind TNF receptor associated factor 6 (TRAF6) and suppress several immune responses (163). Therefore, this induction of HSP70 by celastrol explains its beneficial effects not only in neurodegenerative disorders but also in inflammatory diseases.

In a Parkinson’s mouse model (MPTP neurotoxin induced), it has been shown that celastrol attenuates dopaminergic neurons loss and dopamine concentration deficiency, accompanied by an increase in HSP70 expression and a reduction in inflammation with a decline in TNF and NF-kB production (149). Similarly, in a Huntington rat model (3-NP neurotoxin-induced), studies have demonstrated that celastrol protects from striatal damage and astrogliosis through the induction of HSP70 in dopaminergic neurons *via* heat shock transcription factor (HSF)-1 activation (149, 151). Also, using an experimental model of Parkinson’s disease *in vitro*, Choi et al. have revealed that celastrol protects human dopaminergic cells from injury and apoptosis and prevents ROS generation and mitochondrial membrane potential loss (164). It inhibits cytochrome *c* release, Bax/Bcl-2 alterations, caspase-9/3 activation, and p38 MAPK activation (164). These data propose that celastrol protects dopaminergic cells through the inhibition of mitochondrial-dependent apoptotic pathway and preservation of mitochondria functions, as well as by the inhibition of p38 MAPK activation. Furthermore, using an *in vitro* cadmium-induced model of neurodegenerative diseases, it has been shown that celastrol prevents apoptosis in neuronal cells by inhibition of JNK and Akt/mTOR signaling pathways partly through the increase in their negative regulator PTEN (165). Surprisingly, Konieczny and colleagues have shown in *in vitro* and *in vivo* models of Parkinson disease that celastrol has no neuroprotective effects (166). Authors discuss that it seems to have a narrow therapeutic window, and suggest that it may have a biphasic effect with protective properties at low concentrations and toxic effects at higher concentrations. In accordance, in proteasome inhibitor-lactacystin and mitochondrial toxin-rotenone *in vitro* models of Parkinson’s disease, low concentrations of celastrol are able to partially attenuate cell damage (167). These discrepancies maybe a result of methodological differences among publications, namely cell culture conditions and animal models specificities.

Similar to what is observed in a transgenic mouse model of Huntington disease (151), it has been demonstrated that celastrol reduces the β-amyloid amount in an Alzheimer’s disease model (152). *In vivo* administration of celastrol in a transgenic model of Alzheimer’s disease reduces β-amyloid by inhibiting β-site amyloid precursor protein cleaving enzyme 1 (BACE-1) *via* NF-kB (152). Recently, Chow and co-workers have demonstrated that celastrol induces a set of HSPs (HSP27, 32, and 70) in rat cerebral cortical cultures, which are selectively impacted during the progression of this disease (168). It induces HSP70 in neurons, and HSP27 and HSP32 in glial cells at dosages that do not affect cell viability (169). Additionally, it has been proven *in vitro* that celastrol reduces both LPS-induced cell death and β-amyloid production mainly through the increase in HSP70 but also by increasing Bcl-2 expression and reducing NF-kB, COX-2, and GSK-3β expression and oxidative stress (170). Furthermore, the activated microglia-derived proinflammatory factors, ROS and NO, have long been believed to be involved with neuroinflammation in neurodegenerative diseases, including Parkinson and Alzheimer’s disease (171). Thus, intervention of microglial activation has become an interesting therapeutic target for the treatment of these conditions (172). Of interest, celastrol exhibits anti-inflammatory activity in LPS-activated BV-2 microgial cells through the downregulation of ERK/MAPK phosphorylation and NF-kB activation, which results in the inhibition of inflammatory mediators, such as IL-1β, TNF, and NO (173).

The therapeutic benefits of celastrol have also been elucidated in an amyotrophic lateral sclerosis animal model (G93A SOD1 transgenic mice). It increases mice survival with an augment in neuronal numbers, a reduction in TNF and iNOS levels, and a rise of HSP70 levels in lumbar spinal cord (153). In addition, it has been demonstrated in the experimental autoimmune encephalomyelitis (EAE) mice model that celastrol inhibits pathogenic Th17 cell responses in peripheral LNs (154). This ability of celastrol to interfere with Th17 cells in favor of an anti-inflammatory response was also found in the context of arthritis, as described above. In the EAE mice model, Abdin et al. (155) have further proven that celastrol ameliorates disease signs and relapse and causes a shift in the cytokine profile from Th1 towards Th2 cell pattern. Authors have also shown that it reduces NF-kB expression, nitrites levels, TLR2 expression, and CD3+ T cell count (155).

Celastrol is thus a promising therapeutic candidate for the treatment of neurodegenerative diseases mainly *via* NF-kB and HSPs inhibition. These new highlights in the potential therapeutic applications of celastrol still need intensive investigation due to dosage and toxicological limitations, especially concerning brain-related diseases.

## Therapeutic Properties of Celastrol in Other Diseases

The most recent data regarding the therapeutic applications of celastrol has emerged in diabetes, obesity, atherosclerosis, and hearing loss.

### Diabetes

The exact mechanism underlying type 2 diabetes is still unclear despite the implication of processes such as mitochondrial dysfunction and inflammation (174). In an *in vitro* model of insulin resistance on 3T3-L1 adipocytes with mitochondrial dysfunction, it has been revealed that celastrol markedly improves metabolic functions with a reduction in ROS production and an increase in mitochondrial membrane potential *via* NF-kB pathway inhibition (1). Additionally, in an *in vitro* model of mitochondrial dysfunction and insulin resistance using human skeletal muscle cells, it has been shown that celastrol treatment improves insulin-stimulated glucose uptake activity, apparently *via* PI3K/Akt pathway, with significant enhancement of mitochondrial activities (175). It has also been demonstrated in these cell cultures that it amplifies the expression of AMPK protein and attenuates oxidative damage, PKC θ and NF-kB activation, leading to the reduction of IL-1β, IL-6, and TNF levels (175).

Type 2 diabetes is the leading cause of end-stage renal disease. In this context, it has been recently shown *in vivo* that celastrol treatment not only improved insulin sensitivity and glycemic control but also improved kidney structure and function, through both metabolic and anti-inflammatory effects, possibly *via* NF-kB inhibition (176). Additionally, diabetes often coexists with different metabolic-related syndromes, such as dyslipidemia, hypertension, and liver damage. In a rat model of type 2 diabetes, it has been demonstrated that celastrol reduces macrophage infiltration and downregulates the expression of TLR4, MyD88, and NF-kB, thus decreasing IL-1β and TNF in the hepatic tissue, which can delay the progression of diabetic liver inflammation and injury (177). Another chronic complication of diabetes is the reduction in muscle mass, strength, and physical capacity. Importantly, Guan et al. (178) have shown that celastrol exerts antioxidant effects on skeletal muscle, partly by activating the AMPK/PGC1α/Sirt3 signaling pathway, attenuating diabetic myopathy.

In type 1 diabetes, a study has suggested that celastrol is not effective in reducing disease incidence in NOD mice (179). Using a 25 mg/kg twice a week regimen, celastrol was found to slightly reduce blood glucose levels on the day after dosing, but not at 2 days post administration (179), suggesting that it lowers blood glucose levels acutely. This might also indicate that this is not an effective dose or regimen. In fact, a study of celastrol pharmacokinetics has shown that its half-life is about 10 h in healthy rats (180). Contrarily, an *in vitro* study using RINm5F rat pancreatic β-cell line showed that celastrol regulates cytokine-induced cell death and proinflammatory responses by downregulating iNOS, COX-2, and chemokine (C-C motif) ligand 2 (CCL2) chemokine through NF-kB inhibition, exerting cytoprotective effects (181), which suggests that indeed it may be a therapeutic agent against type 1 diabetes.

### Obesity

In obese condition, hyperleptinemia coexists with the loss of response to leptin, an inhibitor of food intake and inducer of energy expenditure. This phenomenon has been defined as leptin resistance and the restoration of its sensitivity is a useful strategy to treat obesity. Recently, in a hyperleptinemic diet-induced obese mice, celastrol has shown the ability to increase leptin sensitivity (182). It can restore the leptin signaling in neurons by overexpressing anorexigenic peptides pro-opiomelanocortin (POMC) and/or repressing orexigenic peptides [neuropeptide Y (NPY)/AgRP] (182, 183). Also, celastrol ability to increase mitochondrial function and HSF-1, a regulator of energy expenditure, through the activation of a PGC1α-dependent metabolic program in adipose tissues and muscles leads to an augment in energy expenditure and represents a possible therapeutic strategy to treat obesity and its metabolic consequences (184).

### Atherosclerosis

Atherosclerosis is a multifocal, smoldering, chronic immunoinflammatory disease, which pathogenesis involves imbalanced lipid metabolism and a maladaptive immune response that lead to a chronic inflammation of the arterial wall. Importantly, in a rabbit experimental carotid atherosclerosis model, it has been recently shown that celastrol can effectively reduce the plaque ratio, the serum levels of low-density lipoprotein (LDL), and the expression of VEGF, suggesting an antiatherosclerotic effect (185). Another study has also found in a apoE(−/−) mouse fed with a high-fat/high-cholesterol diet that celastrol inhibits lectin-like oxidized LDL receptor-1 (LOX-1) and ROS, preventing atherosclerosis (186). In addition, it has been highlighted that the inhibition of NF-kB pathway was also at least partially involved in this protective effect of celastrol (186). Atherosclerosis is also associated with a dysregulation of endothelial progenitor cells (EPCs). Importantly, *in vitro* and *in vivo* data recently showed that celastrol improves the functional integrity of EPCs, which allows an effective EPC transplantation, used in the treatment of cardiovascular and ischemic diseases (187, 188).

### Hearing Loss

Hearing impairment can be temporary or permanent. It is commonly caused by mechanosensory hair cell death in the inner ear due to aging, noise trauma, chemicals, and therapeutics. Aminoglycoside antibiotics, one of the most used antibiotics, may cause ototoxicity (189). Considering that aminoglycoside-induced hearing loss is irreparable and that its incidence is up to 33% (189, 190), finding a therapeutic strategy has great interest. In this context, it has been recently found that celastrol provides protection against aminoglycoside-induced ototoxicity *in vitro* in utricles and *in vivo* in mice receiving systemic kanamycin, *via* HSP32/HO-1 induction (191). Similar results were also observed in cisplatin-induced ototoxicity (192).

Altogether, the available data suggest that celastrol is a potential therapeutic molecule for the treatment of several chronic conditions but further studies are necessary to substantiate and deepen these conclusions.

## Toxicity and Limitations of Celastrol Formulation

Despite the therapeutic potential of celastrol, further clinical application is still limited by low water solubility, reduced oral bioavailability, narrow window of dosage, and side effects.

Celastrol has poor water solubility (13.25 ± 0.83 mg/ml at 37°C). Its solubility was studied by Qi et al. (193) in various vehicles, and authors have shown that ethyl oleate, olive oil, the surfactants Labrasol and OP-10, and the cosurfactants PEG200, ethanol, butanol, and specially Transcutol P, were adequate solvents for celastrol with a solubility >20 mg/ml.

Due to this poor water solubility, celastrol has low bioavailability. A study from Zhang et al. (180) have demonstrated that oral administration of celastrol in rats results in ineffective absorption into the systemic circulation, with an absolute bioavailability of 17.06%. Li et al. (194). suggest that besides low aqueous solubility *in vivo* metabolism and/or tissue distribution might also cause this poor bioavailability.

One great concern regarding the clinical use of celastrol is its narrow therapeutic window of dose together with the occurrence of adverse effects. Our own data showed *in vivo* that the doses of 2.5 and 5 μg/g/day are effective and non-toxic in the treatment of arthritis in rats; however, lower concentrations immediately lose efficacy and higher concentrations show signs of toxicity (195). The same result has been recently described in Parkinson’s disease models (166). Similarly, in an osteosarcoma xenograft mouse model, it was described that treatment with celastrol at 1 and 2 mg/kg reduced tumor growth (42.9–50.2%), but it caused 5.7–9% weight loss in animals (78). Discrepancies exist in celastrol dosing and toxicity, with data in rodents showing that at 3 mg/kg there are adverse events and 27% mortality but other studies showing no toxic effects at this dose. In addition, there are reports showing an LD_50_ dose of 20.5 mg/kg and others suggesting a 40% mortality at 4 mg/kg (42, 100, 116, 149, 166). One major side effect of celastrol administration might be infertility (29, 196). In fact, a study using mice spermatogenic cells suggests that Ca^2+^ currents inhibition by celastrol may cause antifertility effects (196). In addition, celastrol blocks ion conduction of cardiac Kir2.1 and hERG potassium channels and reduces channel density on cell surface upon chronic treatment (197), which may predict cardiotoxicity. Therefore, new extensive studies on celastrol *in vivo* regimen and toxicity are still needed.

Celastrol may have a dual effect, suppressing oxidative stress at nanomolar concentrations and inducing detectable ROS above 1 μM. Specifically, in contrast to the ROS generation effect of celastrol in cancer cells, some studies have reported that celastrol has antioxidant properties on microglia and endothelial cells and attenuates hypertension-induced oxidative stress in vascular smooth muscle cells (198, 199). This discrepancy may be attributed not only to the difference between non-cancerous cells and cancer cells but also to dosage.

## New Strategies for the Use of Celastrol

To surpass the physicochemical and pharmacokinetic limitations of celastrol and to diminish the effective dose, several methodologies have been tested that can represent useful strategies, such as exosomes (200), lipid nanospheres (201), nanoencapsulation (202), polyamidoamine dendrimer nanocarriers (203), liposomes (204–206), polymeric micelles (207, 208), cell-penetrating peptides-coated nanostructured lipid carriers (194, 209–211), sugar-silica nanoparticles (212), and self-microemulsifying drug delivery system (193). For instance, recent data have shown that celastrol-loaded exosomes enhance free celastrol efficacy and reduce dose-related toxicity in lung cancer (200). Also, celastrol-loaded sugar-decorated mesoporous silica nanoparticles have shown an increased specific anticancer activity with no induced toxicity in HeLa and A549 cells (212). *In vivo*, it has been shown that celatrol-loaded lipid nanospheres, liposomal celastrol, and solid self-microemulsifying dispersible tablets of celastrol significantly increase its oral bioavailability, increase efficacy, and diminishes the occurrence of side effects (193, 201, 205). Although these strategies have demonstrated encouraging results both *in vitro* and *in vivo*, no tripterine preparation have completed the research phase and reached the market.

Depending on the desired therapeutic effect, the concentration range of celastrol is also highly variable. For this reason, it would be interesting to modify its chemical structure producing an effective and less toxic derivative or analog. Some studies have found correlations between celastrol domains and its properties. Structurally, atomic orbital energy of celastrol′s carbons C2 on A-ring and C6 on B-ring possess a great susceptibility towards a nucleophilic attack that gives celastrol antitumor activity against a vast spectrum of tumors (213, 214). Also, it was observed that the celastrol’s acidic carboxylate group was not required for its cytotoxic effect in several cancer cell lines, but instead its quinone methide moiety (215). Different analogs of celastrol were already synthesized, and their neuroprotective properties against t-BHP-induced cytotoxicity were studied in neuronal PC12 cells by Sun and colleagues. Authors have found that the compound CL12 (celastrol coupled with tetramethylpyrazine) is more effective than the parent celastrol (216). In addition, celastrol derivatives with aromatic phenyl substituents appear to inhibit the growth of hepatocellular carcinoma patient-derived xenografts with reduced toxicity (217). Some structure modifications of celastrol were also carried out focusing on either the esterification or amidification of 20-carboxylic acid or the reduction products of A/B rings (215, 216, 218, 219). Structural modifications at the C-2, 3 positions were described as inducers of heat shock response, while those at C-6 position lead to anticancer properties. However, previous studies also suggested that the intact quinone methide moiety was essential for its cytotoxic activity in cancer cells and neuroprotective effect (215, 216, 220).

## Conclusion

Accumulating evidence indicates that the anti-inflammatory, anticancer, and neuroprotective properties of celastrol can be mainly attributed to its ability to inhibit NF-kB, a central player in inflammation, cancer and neurodegenerative diseases. However, it also targets several other molecules, which allows celastrol to have a broad array of pharmacological mechanisms and potential therapeutic applications (Table 1). Celastrol is one of the main components of TW plant that has shown in clinical trials to be efficient in the treatment of different diseases, being generally well-tolerated, but with mild gastrointestinal side effects and possibly fertility issues. Despite the great therapeutic potential of celastrol, there are still some drawbacks in the process of developing it as a new drug. Hopefully, future preclinical studies will provide crucial information regarding celastrol formulation, pharmacokinetics, dosage, and toxicity for further optimization of this compound. The development of new celastrol derivatives and analogs, with higher pharmacological activities and lower toxicological issues, seems to be the next logic step in the development of this therapeutic concept.

**Table 1:** Overview of the mechanisms of action of celastrol in chronic diseases.

Pathology	Pharmacological mechanism	Molecular targets
Inflammatory diseases	Regulation of the production of cytokines, chemokines, and inflammatory mediators	Nuclear factor kappa B (NF-kB), activator protein 1, signal transducer and activator of transcription 3 (STAT3), extracellular signal-regulated kinase (ERK), caspase-1, MD2/TLR4, mitogen-activated protein kinase (MAPK)
	Modulation of inflammatory cell functions	NF-kB, STAT3, hypoxia-inducible factor (HIF)-1α, SYK/MEK/ERK
	Control of bone damage	NF-kB, ERK, c-Fos, transforming growth factor beta-activated kinase 1 (TAK1), receptor activator of nuclear factor kappa-B ligand, mitogen-activated protein kinase (MAPK)
	Antihyperalgesic effect	CB_2_
	Control of intestinal inflammation	Reactive oxygen species (ROS), NLR family pyrin domain containing 3, interleukin (IL)-23/IL-17 axis, E-cadherin, caspase-8, PI3K/Akt/mTOR
	Regulation of lipid metabolism	Stearoyl-CoA desaturase-1 (SCD1)
	Suppression of epithelial mesenchymal transition	FcεRI, protein kinase C (PKC), Rac1, ERK, HSP90

Cancer	Activation of cell death	NF-kB, ROS/c-Jun N-terminal kinases (JNK) pathway, micro-RNAs, androgen receptor, HSP90, HIF/BNIP3, Fas/FasL pathway, mitochondrial respiratory chain complex I, PI3K/Akt/mTOR, AMP-activated protein kinase (AMPK), Erb-B2 receptor tyrosine kinase 2 (ErbB2) and estrogen receptors, caspases, topoisomerase II, AML1-ETO/C-KIT, STAT3/JAK2, Myb, ER stress-eIF2-ATF4-CHOP, GSK3β, proteosomes
	Inhibition of angiogenesis	HIF-1α, HSP90, mTOR/p70S6K/eIF4E pathway, ERK1/2, ROS
	Sensitization to treatment	NF-kB, MAPK, EGFR, ErbB2, surviving, p53, ROS, ATF2/c-JUN, caspase-3, PARP, Bcr-Abl
	Anti-invasive effect	NF-kB, MMP-9, micro-RNAs, Akt, MAPK, Wnt signaling, CXCR4, TAK1, IKK

Neurodegenerative diseases	Inhibition of protein misfolding	HSP, HSF-1
	Protection of dopaminergic neurons	HSP, HSF-1, NF-kB, cytochrome *c*, Bax/Bcl-2, caspase-9/3, MAPK, JNK/Akt/mTOR, PTEN
	Reduction of β-amyloid	NF-kB, HSP, Bcl-2, GSK-3β, ROS

Diabetes	Amelioration of metabolic functions	NF-kB, PI3K/Akt, AMPK, ROS, PKC
	Protection of kidney and liver	NF-kB, TLR4, MyD88
	Reduction of diabetic myopathy	AMPK/PGC1α/Sirt3

Obesity	Induction of leptin sensitivity	Pro-opiomelanocortin (POMC), NPY/AgRP, PGC-1α

Atherosclerosis	Control of lipid metabolism	NF-kB, low-density lipoprotein (LDL), lectin-like oxidized low-density lipoprotein receptor-1 (LOX-1), ROS

Hearing loss	CO production and antioxidant activity	HSP32/HO-1, JNK

## Author Contributions

Drafting manuscript: RC. Revising manuscript content: LFM and JEF. Approving final version of manuscript: RC, LFM and JEF.

## Conflict of Interest Statement

The authors declare that the research was conducted in the absence of any commercial or financial relationships that could be construed as a potential conflict of interest.

## Abbreviations

TW, *Tripterygium wilfordii*; RA, rheumatoid arthritis; CD, Crohn’s disease; CRP, C-reactive protein; ACR, American College of Rheumatology; DMARD, disease-modifying antirheumatic drug; MTX, methotrexate; DM, diabetes mellitus; LPS, lipopolysaccharide; IL, interleukin; PEG2, prostaglandin E_2_; SCW, Streptococcus cell wall-induced arthritis; CIA, collagen-induced arthritis; AIA, adjuvant-induced arthritis; TNF, tumor necrosis factor; NO, nitric oxide; iNOS, inducible nitric oxide synthase; COX, cyclooxygenase; NF-kB, nuclear factor kappa B; AP-1, activator protein 1; IFN-γ, interferon gamma; LN, lymph node; SA, spleen adherent; STAT3, signal transducer and activator of transcription 3; RORγ, RAR-related orphan receptor gamma; Th, T helper; MMP, metalloproteinase; ERK, extracellular signal-regulated kinase; VEGF, vascular endothelial growth factor; Anti-CCP, anti-cyclic citrullinated peptide; RANTES, regulated on activation, normal T cell expressed and secreted; MCP-1, monocyte chemoattractant protein-1; GRO/KC, chemokine ligand 1; MIP-1α, macrophage inflammatory protein 1-alpha; CCR1, C-C chemokine receptor type 1; MD2, lymphocyte antigen 96; TLR, Toll-like receptor; FLS, fibroblasts; MyD88, myeloid differentiation primary response gene 88; HIF, hypoxia-inducible factor; CXCR, C-X-C chemokine receptor; NET, extracellular trap; SYK, spleen tyrosine kinase; MEK, serine/tyrosine/threonine kinase; IkBα, nuclear factor of kappa light polypeptide gene enhancer in B-cells inhibitor, alpha; *SIC*, synovium-infiltrating cells; Treg, T regulatory; RANKL, receptor activator of nuclear factor kappa-B ligand; M-CSF, Macrophage colony-stimulating factor; TAK1, transforming growth factor beta-activated kinase 1; OPG, osteoprotegerin; Trap, tartrate-resistant acid phosphatase; Ctsk, Cathepsin k; NFATc1, nuclear factor of activated T-cells 1; MAPK, mitogen-activated protein kinase; PTH, parathyroid hormone; TRACP5b, tartrate-resistant acid phosphatase 5b; CTX-I, cross-linked C-telopeptide of type I collagen; DPD, deoxypyridinoline; AS, ankylosing spondylitis; PI3K, phosphoinositide 3-kinase; Akt, protein kinase B; CB2, cannabinoid receptor-2; SLE, systemic lupus erythematosus; UC, ulcerative colitis; PBMCs, peripheral blood mononuclear cells; NLRP3, NLR family pyrin domain containing 3; RIP3, receptor-interacting serine-threonine kinase 3; MLKL, mixed lineage kinase domain-like pseudokinase; LITAF, LPS-induced-TNF-α-factor; mTOR, mechanistic target of rapamycin; PCNA, proliferating cell nuclear antigen; EMT, mesenchymal transition; SCD1, stearoyl-CoA desaturase-1; OA, osteoarthritis; HSP, heat shock protein; TIMP, inhibitor of metalloproteinase; FcεRI, Fc epsilon receptor I; PKC, protein kinase C; ROS, reactive oxygen species; JNK, c-Jun N-terminal kinases; AR, androgen receptor; TRAIL, TNF-related apoptosis-inducing ligand; MRC, mitochondrial respiratory chain; AMPK, AMP-activated protein kinase; ErbB2, Erb-B2 receptor tyrosine kinase 2; AML, acute myelogenous leukemia; JAK, Janus kinase; ER, endoplasmic reticulum; PERK, ER stress; eIF2, eukaryotic initiation factor 2; ATF, activating transcription factor; CHOP, C/EBP homology protein; GSK3β, glycogen synthase kinase 3 beta; IP3R, inositol trisphosphate receptor; EGFR, epidermal growth factor receptor; PARP, poly (ADP-ribose) polymerase; IKK, IkappaB kinase; HSF, heat shock transcription factor; NEMO, NF-kappa-B essential modulator; TRAF, TNF receptor associated factor; BACE-1, β-site amyloid precursor protein cleaving enzyme 1; EAE, experimental autoimmune encephalomyelitis; PGC1α, peroxisome proliferator-activated receptor gamma coactivator 1-alpha; Sirt3, NAD-dependent deacetylase sirtuin-3; CCL2, chemokine (C-C motif) ligand 2; POMC, pro-opiomelanocortin; AgRP, Agouti-related protein; NPY, Neuropeptide Y; LDL, low-density lipoprotein; apoE, apolipoprotein E; LOX-1, lectin-like oxidized low-density lipoprotein receptor-1; EPC, endothelial progenitor cell; LD, lethal dose.
